# The Impact of Azathioprine-Associated Lymphopenia on the Onset of Opportunistic Infections in Patients with Inflammatory Bowel Disease

**DOI:** 10.1371/journal.pone.0155218

**Published:** 2016-05-23

**Authors:** Marius Vögelin, Luc Biedermann, Pascal Frei, Stephan R. Vavricka, Sylvie Scharl, Jonas Zeitz, Michael C. Sulz, Michael Fried, Gerhard Rogler, Michael Scharl

**Affiliations:** 1 Division of Gastroenterology and Hepatology, University Hospital Zurich, University of Zurich, Zurich, Switzerland; 2 Division of Gastroenterology and Hepatology, Stadtspital Triemli, Zurich, Switzerland; 3 Zurich Center for Integrative Human Physiology, University of Zurich, Zurich, Switzerland; 4 Division of Gastroenterology and Hepatology, Kantonsspital St. Gallen, St. Gallen, Switzerland; University Hospital Llandough, UNITED KINGDOM

## Abstract

**Background:**

Thiopurines are known to cause lymphopenia (<1,500 lymphocytes/μl). As severe lymphopenia (<500C/μl) is associated with opportunistic infections, we investigated severity of thiopurine-related lymphopenia and development of opportunistic infections in our tertiary referral centre.

**Methods:**

We retrospectively screened medical records of 1,070 IBD patients and identified 100 individuals that developed a total of 161 episodes of lymphopenia during thiopurine treatment between 2002 and 2014. Occurrence of opportunistic infections was documented. A control group consisted of IBD patients receiving thiopurines but without developing lymphopenia.

**Results:**

Of a total of 161 episodes of lymphopenia, 23% were severe (<500C/μl). In this subgroup, thiopurine dosing was modified in 64% (dosage reduction: 32%, medication discontinued: 32%). We identified 9 cases (5.5%) of opportunistic infections, of which only two occurred during severe lymphopenia. One opportunistic infection (4.5%) was identified in the control group. No association was found between opportunistic infections and severity of lymphopenia. All patients who suffered from opportunistic infections were receiving additional immunosuppressive medication.

**Conclusion:**

Our patients treated with thiopurines rarely developed severe lymphopenia and opportunistic infections did not occur more often than in the control group. A careful monitoring of lymphocytes and prophylactic adjustment of thiopurine therapy might contribute to this low incidence.

## Introduction

Azathioprine (AZA) is the best known agent of a family known as thiopurines or purine antimetabolites. AZA was developed approximately fifty years ago and is well established in the treatment of inflammatory bowel disease (IBD) up to the present day, together with its metabolite 6-mercaptopurine (6-MP). It is further being used in other chronic inflammatory diseases, chemotherapy and organ transplant recipients [1–4].

The advantage of AZA/6-MP over placebo for maintenance of remission in Crohn’s disease (CD) has been proven by several meta-analyses showing good evidence for its steroid sparing effect [5, 6]. Due to their slow onset of action, they are usually introduced in combination with fast-acting immunosuppressive drugs, such as steroids that cover the time period until AZA/6-MP takes full effect. Thus, it is conceivable that a recent Cochrane analysis concludes that there is no significant benefit of thiopurine monotherapy for the induction of remission in CD [7–9]. In ulcerative colitis (UC), the available literature displays a similar situation with good evidence for the efficacy of thiopurines for maintenance therapy [10, 11], but not for induction of remission [9, 12].

As an inactive prodrug AZA is converted to 6-MP and further, via multi-enzymatic processing, to 6-methyl-mercaptopurine (6-MMP), 6-thiouric acid (6-TUA) and to active metabolites that can be summarized as 6-thioguanin nucleotides (6-TGN) and 6-methylmercaptopurine ribonucleotides (6-MMPRs). 6-TGN are primarily responsible for both, the cytotoxic potential and the immunosuppressive effect of these agents [13–16]. They are structurally similar to endogenous purine-bases and have a cytotoxic effect on leucocytes by interfering with DNA synthesis [17]. Furthermore, inhibition of the Ras-related C3 botulinum toxin substrate 1 (Rac1) resulting in T-cell apoptosis is presumed [18]. In addition to 6-TGN, 6-MMPRs are also considered to have an immunosuppressive effect by interfering with the synthesis of purine-bases [19, 20]. Eventually, although some details about the mode of action of AZA/6-MP remain unknown to date, it can be concluded that the immunosuppressive effects of thiopurine drugs are mediated by an interplay of various thiopurine metabolites with the cellular and humoral immune system [21–24].

Besides its benefits in the treatment of IBD, AZA is known to potentially cause a broad spectrum of adverse effects, reaching from mild (e.g., itching) to severe (e.g., pancreatitis, liver failure and severe myelosuppression) [22, 25]. Some side effects can be related to specific metabolites, whereas in other cases the exact pathogenesis remains unclear. The hepatotoxic potential for example has been found to be predominantly triggered by 6-MMP and 6-MMPR [16, 26, 27].

In this study, we particularly focus on the development of lymphopenia as a crucial component of myelosuppression. Data on the incidence of AZA related lymphopenia are highly variable, depending on the definition and grading of lymphopenia as well as the study setting, with reported fractions in IBD patients between 10% up to over 30% [28, 29]. In this work, we graduated lymphopenia according to lymphocyte cell counts into mild (1,499–1,000 C/μl), moderate (999–500 C/μl), severe (499–200 C/μl) and most-severe (< 200 C/μl).

Lymphopenia may increase the risk of serious opportunistic infections, as seen in HIV positive patients, primary immunodeficiency, idiopathic CD4+-Lymphopenia and chronic immunosuppressive therapy [30–35]. It has further repeatedly been associated with infectious complications in various chronic inflammatory diseases [33, 36, 37]. Also IBD patients bear a greater risk of opportunistic infections and, according to current literature, AZA/6-MP seem to be at least partially responsible for this [36]. However, data regarding the incidence of infectious complications in patients with IBD or other chronic inflammatory diseases differ considerably, which can, to some extent, be ascribed to different study settings: Data from the TREAT registry, a large North American cohort study, reports serious infections to occur at a rate of 1.7% in patients with CD, regardless of their medical treatment [38]. In a pooled safety analysis of 7 CD and 3 UC cohorts, Lichtenstein et al. counted serious infections in 4.6% of CD and 5.4% of UC (total IBD: 4.9%) patients treated with immunomodulators such as AZA/6-MP or methotrexate (MTX) [39]. When patients with different chronic inflammatory diseases were investigated by Glueck et al. in a German cohort, the rate of infections requiring hospitalisation increased to 13.8% in patients that received AZA/6-MP with or without concomitant steroids [33]. The latter study is of particular interest, as it proved lymphopenia with cell counts < 600 C/μl, respectively CD4+ < 250 C/μl, to significantly increase the risk for development of infections.

Considering that the immunosuppressive properties of AZA are mainly achieved by its cytotoxic effects on T-lymphocytes, it is legitimate to assume that mild to moderate lymphopenia should rather be interpreted as a parameter of a sufficient immunosuppression, than solely as an adverse event.

Therefore, our centre, in accordance with global clinical experience, pursues a policy that tolerates and even targets mild up to moderate lymphopenia but takes prophylactic measures when severe lymphopenia (< 500 C/μl) occurs during treatment with thiopurines. Such prophylactic measures usually include dose reduction or cessation of AZA/6-MP as it has been recommended in earlier studies [25].

In this work, we analysed the occurrence of opportunistic infections in patients with thiopurine-related lymphopenia at the IBD clinic of the Division for Gastroenterology and Hepatology at the University Hospital Zurich.

## Methods

### Patient Selection Process

In this retrospective study we conducted a systematic computer based search for the appearance of the terms ‘azathioprine’, ‘6-mercaptopurine’ or ‘6-thioguanine’ in health records of 1,070 patients from the Swiss IBD Cohort, who were being treated at our clinic between 2002–2014. The records of patients with thiopurines were screened for lymphopenia (defined as < 1,500 lymphocytes/μl) during treatment with this medication. Patients with insufficient documentation of either lymphocyte counts or medication were excluded from analysis. All data was obtained from electronically filed medical reports of the IBD outpatient clinic at the Division of Gastroenterology and Hepatology at the University Hospital Zurich, Switzerland.

### Demographic and Clinical Characteristics

We collected data with regards to patients’ disease and treatment characteristics as well as occurrence of opportunistic infections. Diagnosis of IBD had always been verified by endoscopic and histological examinations. The physician collecting and analysing the data was not involved in patient care. We recorded sex, age, smoking habits and type of disease (CD or UC) including date of first diagnosis. Whenever possible we obtained information about disease characteristics such as the existence of fistulas and/or abscesses, as well as extraintestinal manifestations. In the case of CD, the Vienna Classification was documented. We further noted the course of treatment with purine analogues including date of initiation and cessation, together with dosage and dose-reductions when lymphopenia appeared. Co-medication for IBD (e.g., topical or systemic steroids, 5-aminosalicylic acid containing agents (5-ASA), tumor necrosis factor α inhibitors (TNFα-Inhibitors), methotrexate (MTX), cyclosporine A (CsA), and tacrolimus) was registered separately. Lymphopenia was defined as complete lymphocyte count < 1,500 cells/μl (C/μl) and lowest lymphocyte counts (referred to as nadir) during treatment with AZA/6-MP were graded in 4 groups (mild: 1,499–1,000 C/μl, moderate: 999–500 C/μl, severe: 499–200 C/μl, most-severe: < 200 C/μl). The beginning, duration and recovery of lymphopenia to standard values (> 1,500 C/μl) were assessed chronographically.

We searched for occurrence of opportunistic infections during AZA/6-MP-associated lymphopenia, registered the pathogen, site of infection and its outcome regarding survival. Opportunistic infections were defined as those infections that occur more frequently or solely in patients with a compromised immune system, as e.g. AIDS-defining illnesses [40]. Oral, perianal or vaginal candidiasis was diagnosed by the characteristic clinical appearance and response to therapy with antimycotics. In case of oesophageal candidiasis, endoscopic and histological confirmation was obtained. Diagnosis of CMV colitis was confirmed by either positive CMV IgM, IgG and elevated viral load (CMV DNA >15,000 copies/ml) or by positive PCR for CMV DNA and immunohistochemical detection of CMV infected cells in biopsy specimens.

The same procedure, except for the lymphocyte counts, was applied to the control group which consisted of 22 IBD patients who also received AZA, 6-MP or 6-TG but did not develop lymphopenia. The small sample size of this group was mainly due to inconsistent laboratory records in this retrospective search.

All patients in this study are taking part in the prospective multicentre Swiss IBD Cohort Study and gave written informed consent. The entire data set was anonymized. The study was approved by the local Ethical Committee of the University of Zurich (EK-1316)

### Statistical Analysis

The IBM Statistical Package for the Social Sciences (SPSS, Chicago, IL; version 22) was used to analyse both groups with descriptive statistics. Non-parametric data was expressed as absolute numbers and percentages. Numeric variables were presented as the mean plus standard deviation. A 95% confidence interval and the median with interquartile range were calculated for lymphocyte nadirs. To visualize the distribution of the data we used histograms, boxplots and bar charts. To achieve better readability, percentages displayed in tables were rounded.

## Results

### Demographic Aspects and Disease Characteristics

**Fig 1:**
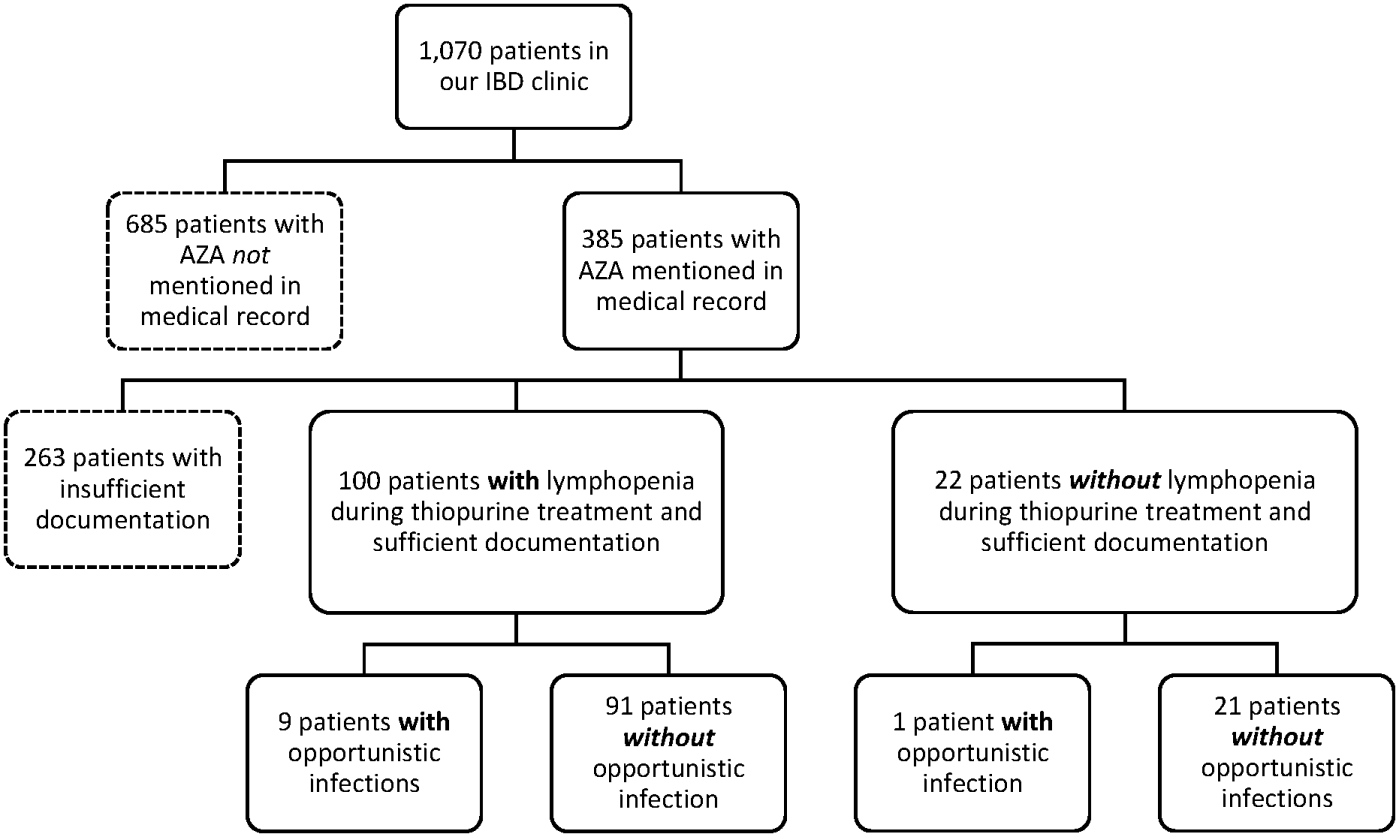
Selection process. This figure shows the patient selection process.

Out of all 1,070 patients in our IBD clinic seen during 2002–2014, 385 patients had AZA or 6-MP mentioned in their medical record at any time (Fig 1). Out of those 385, 263 patients had to be excluded from analysis due to insufficient documentation of either lymphocyte counts or medication. We included 100 patients with IBD (56 with CD, 44 with UC; 48 females) and lymphopenia under AZA/6-MP (71% > 500 C/μl, 29% < 500 C/μl). The control group consisted of the remaining 22 patients, who showed standard lymphocyte values (> 1,500 C/μl) throughout their medical documentation.

Demographic aspects and clinical characteristics are summarized in Table 1. Extraintestinal manifestations were present in 34 (61%) of CD and in 14 (32%) of UC patients. In two (3.6%) CD patients this information remained unknown. With 25 (44.6%) cases, almost half of the patients with CD suffered from fistulae or abscesses. Smoking habits could only be evaluated in 72 (72%) of all subjects. Interestingly, 13 (23.2%) CD patients but only 6 (13.6%) UC patients were smokers. In both groups, 10 (CD: 17.9%, UC: 22.7%) persons quit smoking sometime before development of lymphopenia and 10 (CD, 17.9%), respectively 16 (UC, 36.4%), were non-smokers.

**Table 1:** Patient and disease characteristics.

	Patients with lymphopenia						Patients without lymphopenia (control group)					
	Total		CD		UC		Total		CD		UC	
**Number**	***100***	***100%***	***56***	***56%***	***44***	***44%***	***22***	***100%***	***17***	***77%***	***5***	***23%***
**Female**	48	48%	27	48%	21	48%	11	50%	8	47%	3	60%
**Male**	52	52%	29	52%	23	52%	11	50%	9	53%	2	40%
**Average age at IBD diagnosis** (y)	28	SD: ± 10.68; range: 12.52–56.68	27		30		24		24		24	
**Extraintest. Manifestations**	48	48%	34	61%	14	32%	15	68%	10	59%	5	100%
Unknown	2	2%	2	34%	0	0%	0	0%	0	0%	0	0%
**Abscess/fistula**	25	25%	25	45%	0	0%	6	27%	6	35%	0	0%
Unknown	2	2%	2	4%	0	0%	0	0%	0	0%	0	0%
**Smoking habits:**												
Smoker	19	19%	13	23%	6	14%	3	14%	3	18%	0	0%
Former smoker	20	20%	10	18%	10	23%	3	14%	2	12%	1	20%
Non-smoker	33	33%	10	18%	16	36%	7	32%	4	24%	3	60%
Unknown	28	28%	16	29%	12	27%	9	41%	8	47%	1	20%

Patient and disease characteristics of the lymphopenia and the control group

### Medication

AZA was the most commonly used purine analogue (*n* = 81; 81%), followed by 6-MP (*n* = 16; 16%) and 6-TG (*n* = 3; 3%). At nadir of lymphopenia (lowest lymphocyte count), most patients were receiving additional medication as part of their IBD-treatment. 13 (13%) patients were on sole treatment with purine analogues. In CD, systemic steroids were the most frequently used co-medication whereas 5-ASA was the preferred agent in UC. The average dosage of each type of purine analogue was 138 mg/d (SD: ± 43.14) for AZA, 73 mg/d (SD: ± 19.30) for 6-MP and 30 mg/d (SD: ± 10.00) for 6-TG, respectively.

Thiopurine therapy was stopped in 31 (19%) cases of lymphopenic episodes overall and dosage was reduced during lymphopenia in 28 (17%) patients. When subdivided according to severity of lymphopenia, these numbers become more revealing: Regarding severe lymphopenia (< 500 C/μl), thiopurine was altered in 24 (64%) individuals (dosage reduction: 32%, medication discontinued: 32%). This is more than twice as often as in the group with lymphocytes between 500–1,500 C/μl, in which therapy was reduced in only 16 (13%) and stopped in 19 (15%) patients. Further details regarding medication are displayed in Table 2.

**Table 2:** Medication.

	Total patients		CD		UC		Total episodes		CD		UC	
**Number**	***100***	***100%***	***56***	***56%***	***44***	***44%***	***161***	***100%***	***85***	***53%***	***76***	***47%***
AZA	81	80%	43	77%	38	86%	135	84%	65	76%	70	92%
6-MP	16	16%	10	18%	6	14%	21	13%	15	18%	6	8%
6-TG	3	3%	3	5%	0	0%	5	3%	5	6%	0	0%
**Average dosage** (mg/d):												
AZA	138		131		145		135	SD: ±47.63; range: 50–250	135		135	
6-MP	73		83		58		73	SD: ±22.23; range: 25–100	78		58	
6-TG	30		30		0		26	SD: ±8.94; range: 20–40	26		0	
**Stopped during lymphopenia**	27	27%	19	34%	8	18%	31	19%	20	24%	11	14%
**Dose reduction**	18	18%	9	16%	9	20%	28	17%	15	18%	13	17%
**Co-medication:**												
Anti-TNFalpha	16	16%	13	23%	3	7%	25	16%	20	24%	5	7%
Steroids systemic	57	57%	25	45%	32	73%	76	47%	30	35%	46	61%
Steroids topical	28	28%	15	27%	13	30%	42	26%	19	22%	23	30%
5-ASA	46	46%	10	18%	36	82%	72	45%	14	16%	58	76%
MTX/CsA/Tacrolimus	6	6%	2	4%	4	9%	9	6%	2	2%	7	9%
No co-medication	13	13%	12	21%	1	2%	28	17%	24	28%	4	5%

Medication during lymphopenia displayed separately for all patients and all episodes of lymphopenia.

### Lymphopenia

In the group of 100 patients, 161 episodes of lymphopenia occurred (53% (*n* = 85) patients with CD, 47% (*n* = 76) patients with UC). 32% (*n* = 32) had fluctuating lymphocyte counts and experienced more than one episode of lymphopenia (range: 2–7 episodes). 3 episodes recurred when dose was increased again after a previous reduction and another 3 after foregoing cessation of thiopurine therapy. In 6 cases lymphopenia reappeared when dose was directly increased without having been reduced before. 8 episodes recurred despite dose reduction and no adjustment in thiopurine therapy was made prior to the remaining 42 episodes. The lymphocyte count remained > 500 C/μl in the majority of all lymphopenic episodes (*n* = 124; 77%). The nadir was between 200–499 C/μl in 18.63% (*n* = 30). Most severe lymphopenia (< 200 C/μl) occurred only rarely, accounting for 4.35% (*n* = 7). The two subgroups CD and UC showed a similar pattern of distribution over all four subgroups of lymphopenia levels (1,000–1,499 C/μl, 500–999 C/μl, 200–499 C/μl and < 200 C/μl).

Mild to moderate lymphopenia (1,499–500 C/μl) recovered to standard values (> 1,500 C/μl) more often as compared to those with cell counts < 500 C/μl (100 (81%) mild to moderate vs. 16 (32%) severe to most-severe). 73% (*n* = 37) of mild to moderate lymphopenia recovered spontaneously, but only 44% (*n* = 7) of those with severe to most-severe lymphopenia. Conversely, patients with lymphocyte counts < 500 C/μl recovered more frequently after thiopurine dose reduction (10 (10%) mild to moderate vs. 4 (25%) severe to most-severe) and discontinuation of thiopurine therapy (17 (17%) mild to moderate vs. 5 (31%) severe to most-severe). The remaining 28% (*n* = 45) had not reached standard values again by the latest documented blood examination at the University Hospital Zurich. The exact numbers and further details about the chronological course of lymphopenia are summarized in Table 3.

**Table 3:** Lymphopenia.

	Total episodes of lymphopenia		CD		UC		Thiopurine dose reduction		Thiopurine cessation		Recovery*:		Spontaneously		upon thiopurine dose reduction		upon thiopurine cessation	
**Number**	***161***	***100%***	***85***	***53%***	***76***	***47%***	***28***	***17%***	***31***	***19%***	***116***	***72%***	***80***	***50%***	***14***	***9%***	***22***	***14%***
1'000–1'499 C/μl	63	39%	33	39%	30	39%	6	10%	5	8%	53	84%	44	83%	4	8%	5	9%
500–999 C/μl	61	38%	31	36%	30	39%	10	16%	14	23%	47	77%	29	62%	6	13%	12	26%
200–499 C/μl	30	19%	17	20%	13	17%	12	40%	7	23%	15	50%	7	47%	4	27%	4	27%
< 200 C/μl	7	4%	4	5%	3	4%	0	0%	5	71%	1	14%	0	0%	0	0%	1	100%
> 500 C/μl	***124***	***77%***					***16***	***13%***	***19***	***15%***	***100***	***81%***	***73***	***73%***	***10***	***10%***	***17***	***17%***
< 500 C/μl	***37***	***23%***					***12***	***32%***	***12***	***32%***	***16***	***32%***	***7***	***44%***	***4***	***25%***	***5***	***31%***

Severity and course of lymphopenia.

*Recovery = recover to lymphocyte counts >1,499 C/μl by October 2014 or up to the latest documented blood examination at the University Hospital Zurich

### Opportunistic Infections

Nine patients (5 females; 5 CD, 4 UC) developed an opportunistic infection during lymphopenia. Three patients suffered from CMV colitis and in 4 cases oral and/or oesophageal candidiasis was diagnosed. One patient developed vaginal and perianal candidiasis and one other person had to be treated for herpes zoster (Table 4).

**Table 4:** Site of opportunistic infection.

***Patients with lymphopenia (n = 100)***		
**Organism**	**Site of infection**	**n**
Candida	oesophagus	3
	oral	2
	perianal/vaginal	1
Cytomegalovirus (CMV)	colon	3
Varicella zoster virus (HHV—3)	skin	1
***Patients without lymphopenia (n = 22) (control group)***		
**Organism**	**Site of infection**	**n**
Cytomegalovirus (CMV)	colon	1

Site of infection and pathogen in case of opportunistic infections.

**Fig 2:**
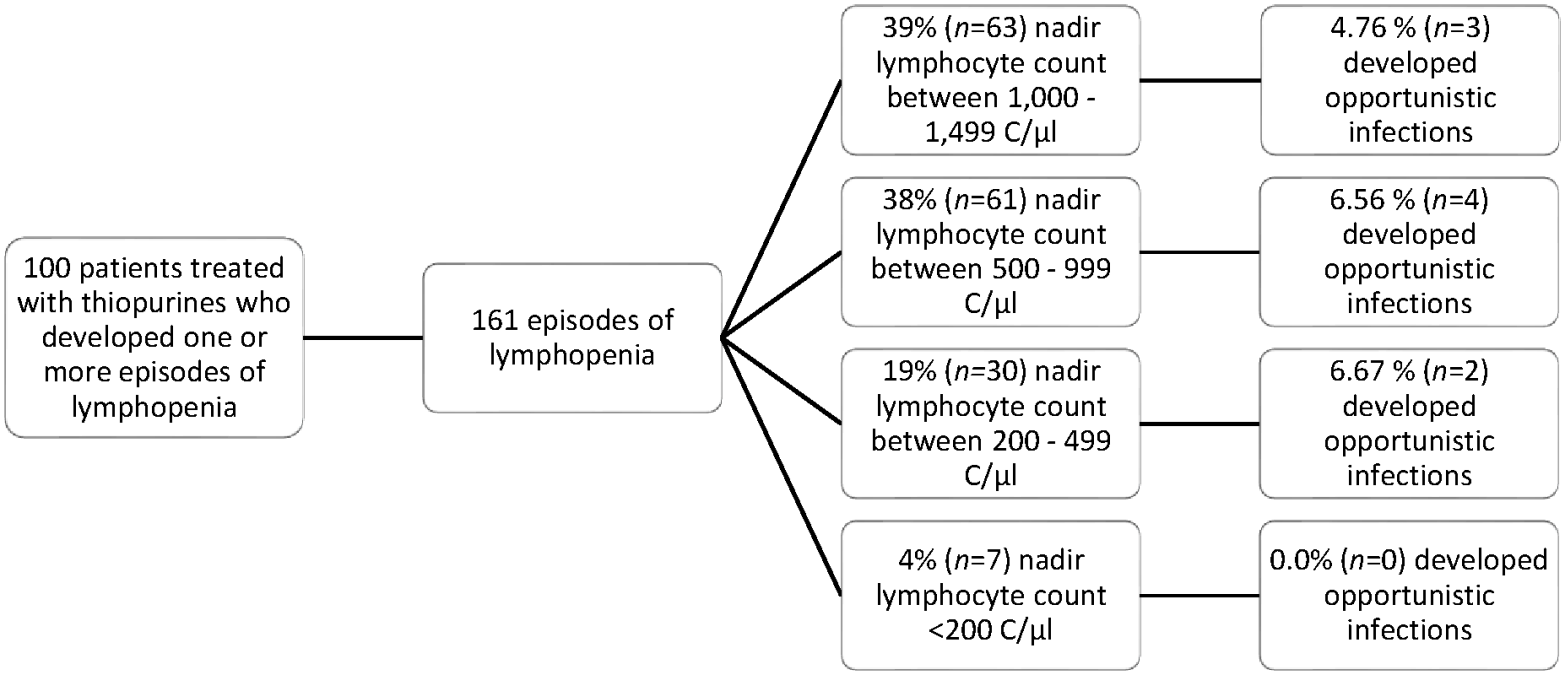
Severity of lymphopenia. Thiopurine-associated episodes of lymphopenia and opportunistic infections, subdivided according to severity of lymphopenia.

The opportunistic infections occurred during lymphopenic episodes with an average nadir of 486 C/μl in CD and 1,120 C/μl in UC. Table 5 shows a broad distribution of lowest lymphocyte counts during opportunistic infections with a wide interquartile range (755 C/μl). Median and mean lymphocyte nadirs lay more than 200 C/μl from each other. Only three out of nine cases did not reach regular lymphocyte counts again by the day of our inquiry or by the end of observation in our hospital. Opportunistic infections were not significantly more frequent (6.67%, *n* = 2 out of 30 episodes) in the subgroup with nadirs between 200–499 C/μl than in the one with 1,000–1,499 C/μl (4.76%, *n* = 3 out of 63 episodes). Opportunistic infections were not observed in the patient group with severe lymphopenia (< 200 C/μl). Fig 2 gives a brief overview of this distribution.

**Table 5:** Lymphopenia during opportunistic infections.

	Opportunistic infections						Total episodes of lymphopenia
	IBD (*n* = 161)		CD (*n* = 85)		UC (*n* = 76)		
**Total**	***9***	***6%***	***5***	***6%***	***4***	***5%***	***161***
1'000–1'499 C/μl	3	5%					63
500–999 C/μl	4	7%					61
200–499 C/μl	2	7%					30
< 200 C/μl	0	0%					7
**Mean duration of lymphopenia (d)**	117						257
**Nadir during opportunistic infection (n = 9)** [C/ul]							
Mean	767		486		1120		
Std. Error of Mean	145						
Median	550						
Std. Deviation	436						
Range	1210						
Minimum	250						
Maximum	1460						
25th percentile	480						
50th percentile	550						
75th percentile	1235						
Interquartile Range	755						

Occurrence of opportunistic infections per lymphopenia subgroup, details about lowest lymphocyte counts (nadir) during opportunistic infection and mean duration of lymphopenia.

Regarding treatment with purine analogues, 7 out of 9 patients received AZA. The mean AZA dosage (171 mg/d) was almost 40 mg higher than the average AZA dosage in all patients with lymphopenia (135 mg/d). One patient each received 6-MP (75 mg/d) and 6-TGN (20 mg/d). AZA/6-MP was reduced or discontinued in only 4 individuals. In two cases this was due to very low lymphocyte counts < 500 C/μl, in one because of CMV colitis and in another one AZA was considered ineffective. In the remaining 5 cases, therapy with purine analogues was continued despite the existence of an opportunistic infection. The average lymphopenic period lasted 428 days (range: 29–907 days) with dosage reduction and 335 days (range: 6–1,072 days) when thiopurines were stopped. Four out of the remaining 5 patients in whom AZA was continued recovered to standard values after a mean of 206 days (range: 3–1,335 days) (Table 6). 6-TGN and 6-MMP levels were measured in 3 out of 9 cases of opportunistic infections. Two individuals had 6-TGN levels within the therapeutic margin (768 and 765 pmol/8*10^8^ Erythrocytes), one showed an insufficient level with 196 pmol/8*10^8^ Erythrocytes. No patient with opportunistic infection had a 6-MMP level above 5,000 pmol/8*10^8^ Erythrocytes.

**Table 6:** Details of opportunistic infections during lymphopenia.

	#1	#2	#3	#4	#5	#6	#7	#8	#9	Summary / Mean
Sex (W = 1, M = 2)	1	1	2	2	1	1	2	1	2	5 W, 4 M
IBD (CD = 1, UC = 2)	1	2	1	1	1	1	2	2	2	5 CD, 4 UC
Age at first diagnosis (y)	45	34	16	48	34	22	14	56	30	33
Extraint. manifestations (1 = yes, 2 = no)	1	2		1	1	1	2	2	2	4
Abscess/fistula (1 = yes, 2 = no)	2	2		2	2	1	2	2	2	1
smoker (1 = yes, 2 = no, 3 = former)	2		3	3	2			3	3	0 S, 2 NS, 4 ExS, 3 unknown
Dosage AZA (mg/d)	175	150	150	150			200	175	200	171
Dosage 6-MP (mg/d)					75					75
Dosage 6-TGN (mg/d)						20				20
AZA/6-MP stop/reduced DURING lymphopenia (1 = stopped, 2 = no alteration, 3 = reduced)	2	3	1	2	3	2	1	2	2	2 stopped, 2 reduced, 5 no alteration
Reason for reduction/stop		lymphopenia	paused because CMV colitis		lymphopenia		no remission achieved			
Co-medication during nadir	Anti-TNFa, MTX/Tacrolimus/CsA	steroids system. & local, 5-ASA	5-ASA	steroids local, MTX/Tacrolimus/CsA	steroids system., 5-ASA	steroids system.	5-ASA	steroids system. & local, 5-ASA	steroids system., 5-ASA	
Nadir during treatment with purine analogue (C/ul)	430	550	540	680	250	530	1410	1060	1460	768
Duration: Start purine analogue—nadir (d)	4807	86		174	180	44	433	452	192	796
Lymphocytes recovery (1 = yes, 2 = no)	2	2	1	1	2	1	1	1	1	6 yes, 3 no
Duration: stop purine analogue—reaching standard values (d)			4				19			12
Duration: nadir—reaching standard values (d)			6	164		55	20	92	7	57
Duration lymphopenia (d)			6	256		320	20	92	7	117
Diagnosis opportunistic infection	Oesophageal candidiasis	Oesophageal candidiasis	CMV colitis and concomitant hepatitis	Oesophageal and oral candidiasis	Oral candidiasis	Herpes zoster	CMV colitis	Vaginal and perianal candidiasis	CMV colitis	

Detailed patient and disease characteristics for lymphopenia-associated opportunistic infections.

All patients who suffered from an opportunistic infection received other medication additionally to AZA/6-MP/6-TGN. Being treated adequately, all subjects fully recovered from their infection.

### Control Group of Patients without Lymphopenia

The control group consisted of 22 patients (11 females, 17 (77%) CD). 6 individuals from the CD subgroup (35%) suffered from an abscessing/fistulising course of their disease, but no one in the UC subgroup did. As regards to use of tobacco among our CD control patients, 3 (18%) declared to be smokers, 2 (12%) were former smokers, 4 (24%) non-smokers and in 8 cases (47%) smoking habits could not be evaluated. No current smokers were detected in the UC group. Here, 3 patients (60%) were non-smokers, one person (20%) stopped smoking and one remained unknown. Patients’ and disease characteristics are summarized in Table 1.

Similar to the lymphopenia group, the majority (86%, *n* = 19) of patients in the control group also received AZA as a purine analogue (mean dosage 175 mg/d, higher dosage in the UC than in the CD subgroup). 14% (*n* = 3) were treated with 6-MP (average dose 67 mg/d) and no one received 6-TGN. The mean duration of therapy with AZA/6-MP was 804 days (Table 7).

**Table 7:** Control group.

	Total		CD		UC	
**Number**	***22***	***100%***	***17***	***77%***	***5***	***23%***
**Medication**						
AZA	19	86%	15	88%	4	80%
6-MP	3	14%	2	12%	1	20%
6-TGN	0	0%	0	0%	0	0%
**Average dose (mg/d):**						
AZA	139	SD: ±35.59; range: 50–200	129		175	
6-MP	67	SD: ±14.43; range: 50–75	63		75	
6-TGN	0	SD: ±8.94; range: 20–40	0		0	
Unknown	1		1		0	
**Mean duration of therapy with purine analogues (d)**	804		803		810	
***Opportunistic infection*:**	***1***	***5%***	0	0%	1	20%
Dosage AZA (mg/d)	200					
Co-medication during opport. Infection	steroids syst. and local, 5-ASA					
Diagnosis opport. infection	CMV colitis, 10.02.2012					

Details about thiopurine therapy and occurrence of opportunistic infections in the control group.

Of all 22 cases, one (5%) developed an opportunistic infection: One male UC patient suffered from CMV colitis while being treated with AZA 200 mg/d for 639 days. During the infection he received co-medication with topical and systemic steroids and a 5-ASA preparation.

## Discussion

In this retrospective search we investigated the occurrence of thiopurine related opportunistic infections in IBD patients and found no association between opportunistic infections and severity of lymphopenia, while prophylactic adjustment of medication might have contributed to this result.

In our study, opportunistic infections were of mostly mild character (e.g. muco-cutaneous candidiasis, herpes zoster) and occurred in only 5.6% of all patients with lymphocyte counts < 1,500 C/μl. When calculated for severe lymphopenia only (< 500 C/μl) the frequency even dropped to 1.24%, which is astonishingly low, compared to the studies mentioned above [33, 39]. The incidence further decreased when CMV colitis, an infection that is debatable to be counted as an opportunistic infection, was excluded. In our cohort, CMV colitis occurred equally often in patients with lymphopenia as in the control group. To date, to the best of our knowledge, no precise data regarding overall prevalence of CMV reactivation in IBD exist, to which we could compare our patient collective. Most of the reports that exist on this topic included only a narrowed patient group or used different diagnostic criteria [41].

Lymphopenia has mainly been attributed to exceeding 6-TGN levels [42–44]. These metabolites accumulate in the tissue and can be measured in erythrocytes, but its widespread use for drug monitoring is currently a point at issue and needs to be further evaluated [9, 45]. The intracellular concentration depends on many factors, such as dosage and inter-individually highly variable bioavailability of AZA/6-MP, enzyme activity of the thiopurine-methyltransferase (TPMT) and interactions with other drugs. Levels exceeding the therapeutic margin can lead to severe myelosuppression with potentially lethal septic complications [46–52]. As 6-TGN is also considered to primarily mediate the immunosuppressive effects of AZA, a narrow therapeutic margin has to be presumed. Therefore the pharmacodynamics of AZA suggest that mild lymphopenia might be proof of a sufficient immunosuppression with therapeutic intracellular concentration of 6-TGN, whereas severe lymphopenia and its consequences result from excessive levels of the latter [53]. It is consequently of crucial importance to differentiate between mild to moderate (1,499–500 C/μl) and severe lymphopenia (<500 C/μl) when interpreting data about lymphopenia.

As in our data, opportunistic infections were not more frequent in case of severe lymphopenia. One possible interpretation is that thiopurine induced lymphopenia might not be as immune compromising as lymphopenia in other medical conditions, such as e.g. advanced HIV infection. However, we believe that prophylactic therapy adjustment, as is was often made in our collective with severe lymphopenia, could have substantially contributed to such a low incidence of opportunistic infections in this subgroup. It may also be a reason for the comparatively few patients with cell counts < 200 C/μl, as a threshold of 500 C/μl for prophylactic dosage adjustment might have stopped a further decline. Additionally, other authors have described severe lymphopenia to be associated with infectious complications. Glueck et al. for example identified in a German cohort total lymphocyte counts < 600 C/μl and CD4+ lymphocyte counts < 250 C/μl as individual risk factors for the development of infections requiring hospitalisation (not limited to opportunistic infections) in patients with chronic inflammatory diseases, irrespective of their immunosuppressive therapy [33].

Regarding therapy adjustments, it is not surprising that both reduction and cessation were made more frequently in patients with severe to most-severe lymphopenia. However, as no official recommendation for the adjustment of thiopurine therapy exists, no adaption of dosage was made in a significant amount of the cases with severe lymphopenia. The decision whether to reduce thiopurine dosage or not is left to the treating physician’s discretion and dependent not only on the absolute lymphocyte count, but also on diseases severity, course of disease, co-medication and many other factors. The adjustments made in the collective with only mild to moderate lymphopenia may have mainly been due to reasons other than lymphopenia.

It needs to be considered that lymphocyte counts are also influenced by many other variables such as physical or psychological stress, co-medication, disease activity and concomitant diseases. Especially viral infections can considerably influence lymphocyte counts and susceptibility to infections. Additionally, aging related attenuation of immune-competence is of importance [54–58].

Particular emphasis has to be laid on co-medication, as all patients with infections during lymphopenia received additional immunosuppressive medication. Combined immunosuppression is likely to bear a greater risk not only for development of lymphopenia [28] but also for opportunistic infections [33, 36]. For CMV re-activations specifically, an increased risk for active infection in patients with double immunosuppression consisting of AZA and TNFα-inhibitors was described [59]. Furthermore, systemic or local steroids bear a risk for development of mucocutaneous candidiasis [36]. Systemic steroids were the most frequent co-medication among patients with opportunistic infections (as well as in the whole study population) and could explain the high number of mucocutaneous candidiasis in this group. Thus, our findings support the growing evidence that a combined immunosuppression in patients with IBD increases the risk for opportunistic infections.

Eventually, the difficulty for the physician in case of lymphopenia during thiopurine treatment is to adapt therapy in a way that achieves a remission maintaining immunosuppressive level but does not drastically increase the risk for infectious complications due to myelosuppression. Therefore, a regular blood count monitoring is of major importance and cannot be replaced by other prophylactic measurements like TPMT genotyping or metabolite monitoring [9, 60, 61].

Our study has weaknesses and strengths. A strength is the fact that health records for IBD outpatient and inpatient consultations at the University Hospital of Zurich are standardized, which results in a homogenous data quality. Furthermore, our study analysed a cohort which is representative for a characteristic Western IBD patient collective. The fact that it was conducted at a tertiary university hospital can be interpreted as an advantage as well as a disadvantage. As difficult cases such as opportunistic infections in IBD patients tend to converge in tertiary centres, the probability was high that such cases would have been registered in our centre. This on the other hand could reflect a selection bias that overestimates the incidence of opportunistic infections among patients with lymphopenia. Accordingly, the low rate of severe infection even in sever lymphopenia appears somewhat reassuring.

Several methodological limitations of the data presented need to be considered. The retrospective study design substantially limited the number of patients that could be included: Laboratory results for lymphocyte counts were partially incomplete, due to intermittent observation at our centre. This could result in an overestimated duration of lymphopenic episodes (date of first value < 1,500 C/μl—date of recovery to a value > 1,500 C/μl). Serum levels of 6-TGN and 6-MMP in particular, were only recorded inconsistently and could therefore not be correlated with lymphopenia or opportunistic infections. IBD patients in remission, with mild flares or mild opportunistic infections such as oral thrush are often managed by district hospitals, by gastroenterologists in private practices or by general practitioners. The consecutive small sample size of our control group did not allow us to match for sex and age or to apply comparable statistics. A prospective study design would enable to measure T-cell subsets instead of total lymphocyte counts, as these were not determined in standard laboratory monitoring. Furthermore, studies conducted at tertiary centres entail a selection bias for patients with active flairs and more severe disease. The high percentage of patients treated with steroids in our collective could be a reflection of this.

The sheer variety of influencing factors in IBD patients makes a bias-free evaluation of risk factors extremely complex and costly. Still, given the clinical relevance, it would be interesting to look at some of them in more detail: As Glueck et al. identified CD4+ lymphocyte counts to be a better predictor for infectious complications than total lymphocyte counts [33], evaluating the influence of AZA on the distribution of T-lymphocyte subtypes in future studieswould enable direct comparison with AIDS patients.

Another point of interest is that an increasing incidence of *Pneumocystis carinii* pneumonia (PCP) in IBD has been reported and combined immunosuppressive therapy or lymphocyte counts < 400 C/μl or <300 C/μl have been suggested as risk factors [62, 63]. Even though antimicrobial PCP prophylaxis has been proven to be highly effective in HIV negative immunocompromised patients [64], there is still no clear evidence for PCP prophylaxis in IBD [62].

In summary and having regard to recent studies, persistent severe lymphopenia (lymphocyte count < 500 C/μl) might expose the patient to a greater risk for infectious complications. Close lymphocyte monitoring and prophylactic dose adjustments in case of thiopurine-induced severe lymphopenia may be recommended, as this procedure is likely to reduce the number of infectious complications in these patients. However, when this careful monitoring and consequent dose adjustment is performed, the risk for infectious complications might be substantially lowered, maybe even to the risk of those patients not receiving thiopurine therapy. Further prospective studies are needed to confirm our assumption and to define an exact cut-off lymphocyte value.
